# Influence of Trace Magnesium Addition on Inclusion Modification and Localized Corrosion Behavior of 304 Austenitic Stainless Steel

**DOI:** 10.3390/ma19214528

**Published:** 2026-10-27

**Authors:** Faiqa Barkat, Lei Huang, Marina Afzal, Sundas Arshad, Aiman Mukhtar, Swaira Arshad, Qing Li, Kaiming Wu

**Affiliations:** 1 State Key Laboratory of Refractories and Metallurgy, Hubei Province Key Laboratory of Systems Science in Metallurgical Process, International Research Institute for Steel Technology, Collaborative Innovation Center for Advanced Steels, Wuhan University of Science and Technology, Wuhan 430081, China; faiqabarkat11@gmail.com (F.B.); afzalmarina786@gmail.com (M.A.); arshadsundas@wust.edu.cn (S.A.); swairaarshad18@gmail.com (S.A.); 13797235225@163.com (Q.L.); 2 Guangxi BeigangNew Materials Co., Ltd., Beihai 536000, China

**Keywords:** austenitic stainless steel, magnesium addition, inclusion modification, pitting corrosion, SKPFM

## Abstract

Non-metallic inclusions are preferential sites for pitting corrosion initiation in austenitic stainless steels. This study investigates the effect of magnesium addition on inclusion evolution and localized corrosion behavior of austenitic stainless steel using SEM–EDS, immersion testing, scanning Kelvin probe force microscopy (SKPFM), potentiodynamic polarization, and electrochemical impedance spectroscopy (EIS). Magnesium addition modifies the inclusion population from coarse (Mn,Al)-O into finer MgO·Al_2_O_3_ spinel-based inclusions with improved distribution. Immersion testing in FeCl_3_–HCl solution reveals delayed inclusion dissolution and reduced localized pitting attack in the Mg-treated steel. SKPFM maps, together with immersion observations, provide qualitative evidence of changes in the local electrochemical contrast between inclusions and the surrounding matrix. Electrochemical measurements in 3.5 wt.% NaCl further demonstrate improved corrosion resistance after Mg addition, as evidenced by an increase in pitting potential from 0.28 V to 0.52 V vs. SCE and charge-transfer resistance. The improved corrosion resistance is associated with inclusion refinement and modification of the local surface-potential distribution, which may influence inclusion-related localized corrosion processes. Overall, Mg microalloying modifies the inclusion characteristics of austenitic stainless steel and enhances its resistance to chloride-induced localized corrosion, demonstrating the potential of inclusion engineering as a strategy for improving stainless-steel corrosion performance.

## 1. Introduction

Austenitic stainless steels are widely used in marine engineering, the petrochemical and chemical processing industries, biomedical devices, and structural applications due to their excellent combination of mechanical strength, toughness, weldability, and corrosion resistance. Among these, AISI 304 has attracted considerable industrial interest because of its good mechanical properties and superior resistance to localized corrosion [1,2,3,4]. These desirable properties originate primarily from the spontaneous formation of a thin, compact, and self-healing Cr-rich passive film on the steel surface, which acts as an effective barrier against aggressive environments [5,6].

Despite this inherent passivity, 304 stainless steel remains susceptible to localized corrosion in chloride-containing environments. Pitting corrosion is particularly critical because of its autocatalytic propagation, metastable to stable transition behavior, and potential to cause sudden catastrophic failure without significant overall material loss [7]. It is now well established that pitting initiation is strongly influenced by microstructural heterogeneities, especially non-metallic inclusions [8]. Among these, MnS inclusions are widely recognized as preferential sites for localized corrosion because their dissolution and the associated inclusion/matrix interfacial processes can promote pit initiation. Consequently, inclusion characteristics such as size, morphology, chemistry, and spatial distribution are critical factors governing localized corrosion susceptibility [9,10].

To mitigate inclusion-induced corrosion, considerable effort has been devoted to inclusion engineering through secondary metallurgy and microalloying strategies. Calcium treatment is widely adopted to modify sulfide morphology and improve steel cleanliness; however, excessive Ca addition can lead to the formation of coarse CaS-rich inclusions, nozzle clogging, and deteriorated castability [11,12]. Rare-earth element modification can effectively regulate inclusion chemistry because of the strong affinity of these elements for oxygen and sulfur [13]; nevertheless, their industrial application is limited by high cost, process instability, and the formation of thermodynamically stable oxides and sulfides [14]. These limitations highlight the need to develop more efficient, controllable, and industrially viable microalloying strategies for inclusion regulation.

In this context, magnesium has emerged as a promising microalloying element for inclusion modification. Mg can effectively transform Al_2_O_3_ inclusions into Mg-containing oxides such as MgAl_2_O_4_ spinel, leading to refined inclusion size and improved distribution uniformity [15]. Jiang et al. found that Ca-Mg microalloying transformed MnS-Al_2_O_3_ inclusions into refined (Ca,Mn)S-MgO-Al_2_O_3_ composite inclusions, which reduced local strain concentration and modified the localized corrosion process [16]. Similarly, Wei et al. demonstrated that an appropriate Mg addition significantly enhanced the corrosion resistance of 310S stainless steel, whereas excessive Mg adversely affected the integrity of the protective surface scale [17]. Li and Shi also showed that magnesium treatment transformed Al_2_O_3_ into MgAl_2_O_4_ spinel phases, leading to a more homogeneous inclusion distribution [18].

Nevertheless, despite increasing interest in magnesium-treated stainless steels, the fundamental relationship between Mg-induced inclusion modification, local electrochemical heterogeneity, and chloride-induced pitting initiation remains unclear at the microelectrochemical level. In particular, the coupling between inclusion characteristics, inclusion–matrix Volta potential differences, localized corrosion evolution, and the overall electrochemical response requires further clarification. Moreover, the relationship between Mg-induced microstructural modification and the chemistry of the surface passive film remains insufficiently understood.

Therefore, the novelty of this work lies in elucidating the multiscale relationship between Mg-induced inclusion modification and localized corrosion of 304 stainless steel, with particular emphasis on the linkage between inclusion/matrix electrochemical heterogeneity, inclusion-associated dissolution, passive-film chemistry, and macroscopic pitting behavior. In this work, the influence of Mg addition on the inclusion characteristics and localized corrosion behavior of 304 stainless steel was systematically investigated using SEM–EDS, time-resolved immersion testing, scanning Kelvin probe force microscopy (SKPFM), potentiodynamic polarization, electrochemical impedance spectroscopy (EIS), and X-ray photoelectron spectroscopy (XPS). The inclusion evolution, local Volta potential distribution, and corrosion morphology were correlated with the macroscopic electrochemical response and passive-film chemistry. By integrating these observations across microstructural, local electrochemical, and macroscopic scales, this study clarifies how Mg-induced inclusion modification affects localized corrosion initiation and the subsequent corrosion resistance of 304 stainless steel in chloride-containing environments.

## 2. Materials and Methods

### 2.1. Sample Preparation

Two 304 austenitic stainless-steel variants were investigated: one in the as-received condition (Mg-free) and the other modified by magnesium addition (Mg-treated). The Mg-free and Mg-treated steel variants each originated from a separate industrial heat supplied by Guangxi Beihai New Materials Co., Ltd., **Beihai, China**, with one heat representing each variant. They were fabricated using electric arc furnace melting and subsequently subjected to secondary refining processes, argon oxygen decarburization (AOD) and ladle furnace (LF), to achieve the targeted chemical composition and lower impurity levels. Magnesium was added during the ladle furnace refining stage in the Mg-treated steel. After the addition, the melt was gently stirred under argon protection for at least 10 ± 0.5 min without exposure to air, ensuring effective interaction between magnesium and inclusions as well as compositional uniformity. The refined steels were cast into slabs and then hot worked in the temperature range of 1100–1250 °C with a temperature uncertainty of ±10 °C to obtain the required geometry and promote microstructural homogenization. To eliminate pre-existing carbide phases and obtain a homogeneous austenitic microstructure, the alloys were solution-annealed at approximately 1050 ± 5 °C and subsequently quenched in water. The elemental compositions of the steels were analyzed by inductively coupled plasma atomic emission spectroscopy (ICP–AES, iCAP 7400, Thermo Fisher Scientific, Waltham, MA, USA)), with the corresponding results summarized in Table 1. For each steel variant, one specimen was used for sequential SEM–EDS and SKPFM characterization and immersion testing to track the evolution of the same inclusions. Three specimens were prepared for electrochemical measurements, and one additional specimen was used for XPS analysis.

For electrochemical testing, specimens measuring (10 ± 0.1) × (10 ± 0.1) mm were sectioned from (5 ± 0.1) mm-thick steel plates using wire electrical discharge machining (WEDM, FA10S, Mitsubishi Electric Corporation, Tokyo, Japan). This cutting method was selected to ensure accurate specimen geometry while avoiding excessive thermal damage to the material surface. A copper wire was attached to each sample to provide electrical connection, after which the specimens were encapsulated in epoxy resin with only the designated test surface left uncovered. Surface preparation was carried out by sequential grinding with SiC papers (100–2000 grit), followed by final polishing with a fine diamond abrasive suspension ~1.5 μm, yielding a highly smooth and reflective surface. After the final polishing step, the samples were thoroughly cleaned with ethanol and air-dried to remove polishing residues and possible surface contaminants. Before electrochemical testing, the prepared specimens were stored in a desiccator to minimize surface contamination and moisture exposure.

### 2.2. Characterization of Surface Morphology

The morphology, distribution, and elemental characteristics of non-metallic inclusions were investigated using a field-emission scanning electron microscope (FE-SEM, AURIGA, Zeiss, Oberkochen, Germany) coupled with an energy-dispersive X-ray spectroscopy (EDS, Genesis 2000, EDAX, Mahwah, NJ USA) system. Microstructural observations were conducted in secondary electron imaging mode at an accelerating voltage of 15 ± 0.1 kV with a working distance of 10 ± 0.2 mm. Quantitative inclusion analysis was performed using the AZtecSteel 6.3 automated characterization platform. For each alloy, one specimen was examined, and 48 observation regions were randomly selected for analysis. A total of 2300 inclusions were detected in the Mg-free steel and 1205 in the Mg-treated steel, with a scanned area of approximately 10 mm^2^ for each alloy. The automated analysis provided information on inclusion population, size distribution, and chemical composition. Under the selected operating parameters, inclusions as small as approximately 0.4 μm could be detected and characterized [19]. Inclusions were classified based on their elemental composition determined by EDS mapping using the following criteria: (i) oxide: O ≥ 10 at.% and a metal-to-O ratio consistent with oxide stoichiometry; (ii) sulfide: S ≥ 10 at.% and Mn ≥ 10 at.%; (iii) oxysulfide/composite inclusion: both O-rich and S-rich regions coexist within a single particle; and (iv) particles smaller than 0.4 μm were excluded, consistent with the detection limit of the automated analysis.

### 2.3. Immersion Corrosion Test

Short-term immersion testing at 25 ± 0.5 °C was performed to clarify how non-metallic inclusions govern the initial stages of pitting degradation. Specimens were exposed to the corrosive medium for different durations: 0 s, 4 min, 10 min, and 18 min, with the immersion times monitored using a smartphone stopwatch. At each predetermined immersion timepoint, samples were extracted from the solution, rinsed with deionized water, and subsequently dried. The corrosive immersion medium was prepared by first dissolving 69.9 ± 0.1 g of ferric chloride hexahydrate (FeCl_3_·6H_2_O) within 350 ± 2 mL of deionized water. After complete dissolution of the iron salt, 20 ± 0.5 mL of concentrated hydrochloric acid (mass fraction: 36–38 wt.%) was added into the aqueous mixture [20]. To enable repeated observation of identical inclusions throughout the corrosion process, reference marks were created on the specimen surface prior to testing. The relative positions between these marks and selected inclusions were measured and documented in advance, allowing the same regions to be precisely relocated during subsequent microscopic examinations. By combining this relocation method with SEM observations, the progressive evolution of inclusion-induced localized corrosion could be continuously monitored at different immersion stages. In addition, the elemental composition of inclusions and surrounding corrosion products was examined by EDS analysis. Five inclusions were monitored for each steel variant during immersion testing. Two representative inclusions from each variant are presented in Figures 4 and 5, respectively.

### 2.4. Electrochemical Corrosion Characterization

All electrochemical characterizations were conducted at ambient temperature in a standard three-electrode electrolytic cell setup. A Zahner Zennium potentiostat (Zahner Elektrik GmbH & Co. KG, Kronach, Germany) served as the testing platform, with an aqueous 3.5 wt.% sodium chloride solution used as the corrosive electrolyte under naturally aerated conditions. The steel specimen served as the working electrode, with an exposed surface area of 1.0 ± 0.02 cm^2^. A Shanghai Leici 260-type platinum-black conductivity electrode(INESA Scientific Instrument Co., Ltd., Shanghai, China) was used as the counter electrode, while a saturated calomel electrode (SCE) (INESA Scientific Instrument Co., Ltd., Shanghai, China) served as the reference electrode. Before testing, the exposed surface was mechanically polished to a mirror-like finish following the previously described preparation procedure. To remove the air-formed surface film and establish a consistent initial surface condition, each specimen was subjected to pre-test potentiostatic polarization at −0.8 ± 0.002 V for 900 s [21]. After monitoring the open-circuit potential (OCP) for 1 h, electrochemical impedance spectroscopy (EIS) measurements were performed using an AC perturbation amplitude of 5 ± 0.1 mV over a frequency range of 100 kHz to 0.01 Hz (instrumental uncertainty ±0.1%). Potentiodynamic polarization tests were subsequently performed at a scan rate of 0.3 ± 0.01 mV s^−1^, over the potential range from −0.5 ± 0.002 V to 1.0 ± 0.002 V vs. SCE. While lower scan rates such as 0.16 mV s^−1^ are widely adopted for potentiodynamic polarization measurements to minimize scan-rate-related distortion, a scan rate of 0.3 mV s^−1^ has also been documented for 304 stainless steel in 3.5 wt.% NaCl solution using an SCE reference electrode [22]. Under the present experimental conditions, no significant scan-rate-induced distortion of polarization curves was observed. This scan rate was kept identical for all tested specimens to enable direct comparative assessment of corrosion responses among samples. Key corrosion parameters, including corrosion current density, pitting potential and passive current, were determined from the polarization curves. Tafel extrapolation for i_corr_ was performed by drawing linear tangents along the straight cathodic and anodic Tafel segments in Origin 2023. The pitting potential E_p_ was defined as the potential at which the anodic current density exhibited a sharp and sustained increase from the passive current region.

The collected EIS datasets were analyzed using ZSimpWin software (version 3.6). The experimental impedance results were compared with the simulated data obtained using an equivalent electrical circuit and complex nonlinear least-squares (CNLS) fitting [23,24]. The chi-squared (χ^2^) value was used to evaluate the fitting quality between the experimental and simulated impedance data. Each electrochemical measurement was performed at least three times, and the results are presented as mean ± standard deviation.

SKPFM measurements were performed using a Bruker AFM equipped with a conductive SCM-PIT-V2 probe consisting of antimony-doped silicon with a PtIr coating. The probe had a nominal spring constant of 3 N m^−1^ and a resonance frequency of 75 kHz. Measurements were conducted at room temperature in ambient air, using enhancement mode with a lift height of 90 ± 3 nm. The scan rate was set to 1 Hz, and the images were acquired at a resolution of 256 × 256 pixels. The measured Volta potential distribution was used to compare the local surface-potential contrast between non-metallic inclusions and the surrounding steel matrix.

To characterize the passive film, a passive layer was first formed on the steel surface by applying a potentiostatic polarization at 0.1 ± 0.002 V for 5400 s in 3.5 wt.% NaCl solution. Subsequently, the passive film was characterized using an X-ray photoelectron spectrometer (AXIS SUPRA+, Kratos Analytical, Manchester UK) and photoelectrons were excited using a monochromatic Al Kα X-ray source. The XPS data were analyzed using Avantage software(Version 5.9915) to determine the composition and proportions of various compounds within the passive film. Curve fitting was performed using Gaussian-Lorentzian functions with Shirley background subtraction. The binding energy scale was calibrated using the C 1s peak at 284.8 eV. Peak positions were held at identical binding energies for both the Mg-free and Mg-treated samples, and the FWHM of corresponding components was limited to ±0.3 eV to ensure comparability. The peak-fitting parameters (binding energy, FWHM) for all components are summarized in Table S1. One representative specimen was analysed for each steel (Mg-free and Mg-treated).

### 2.5. Thermodynamic Calculations

Thermodynamic equilibrium simulations were performed using Thermo-Calc 2023a to predict the stability and transformation behavior of complex oxide and sulfide inclusions. The TCFE12: Steels/Fe-Alloys v12.0 thermodynamic database was used. The full measured chemical compositions of experimental steels (including oxygen content) given in Table 1 were used as input. A one-axis equilibrium calculation was conducted, in which the temperature was linearly scanned from 500 K to 1600 K with a step size of 50 K at a constant pressure of 100,000 Pa (1 bar) and a fixed system size of 1 mol. All relevant oxide and sulfide phases were activated in the phase constitution for the equilibrium calculation.

## 3. Results

### 3.1. Two-Dimensional SEM-EDS Characterization of Complex Inclusions

**Figure 1:**
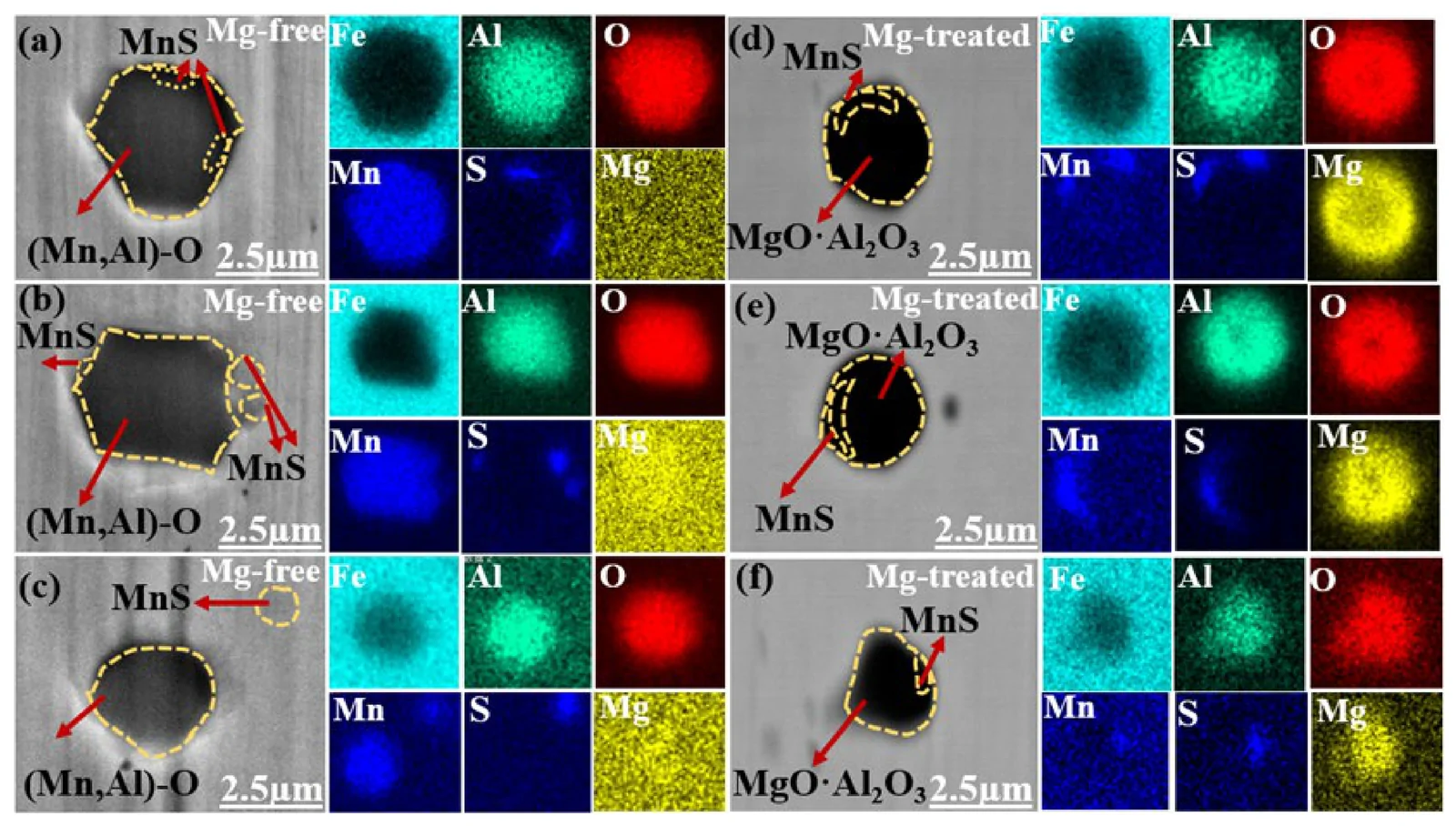
Morphologies and EDS elemental maps of representative (Mn, Al)–O and MnS inclusions observed in Mg-free steel (**a**–**c**); corresponding results for typical MgO·Al_2_O_3_ and MnS inclusions in the Mg-treated steel (**d**–**f**). Yellow dotted lines outline inclusion boundaries and red arrows mark inclusions.

Scanning electron microscopy paired with energy-dispersive X-ray spectroscopy elemental mapping was used to analyze the shape and elemental composition of impurity inclusions in Mg-free and Mg-treated steels; representative micrographs are displayed in Figure 1. Significant variations in inclusion geometry, chemical constitution, and microstructural features were observed after magnesium addition, demonstrating the strong modification effect of Mg treatment on the inclusion population within the steel matrix. In the Mg-free steel (Figure 1a–c), the inclusions are mainly characterized by relatively large sizes, irregular morphologies, and chemically heterogeneous structures. Elemental mapping indicates that these inclusions are primarily enriched in Mn, Al, and O, corresponding to complex (Mn,Al) oxide inclusions. Furthermore, localized enrichment of Mn and S appears at the edges of each inclusion, implying the formation of manganese sulfide along the boundary separating oxide inclusions and the steel matrix.

In direct comparison, the inclusions in the Mg-treated steel (Figure 1d–f) exhibit obvious refinement, improved dispersion, and a more spherical morphology. After magnesium addition, the original (Mn,Al)-oxide inclusions are transformed into thermodynamically stable Mg-containing oxides, predominantly MgO·Al_2_O_3_ inclusions. Although MnS is still detected in the Mg-treated steel, it mostly precipitates along the boundaries of the newly modified MgO·Al_2_O_3_ inclusions, forming oxide–sulfide composite inclusions. Compared with the inclusions in the Mg-free steel, the Mg-modified inclusions exhibit improved morphological regularity and reduced size. These results suggest that magnesium addition not only changes the inclusion chemistry and morphology but also alters the interfacial characteristics of MnS, which may subsequently influence the localized electrochemical behavior and corrosion response of the steel.

**Figure 2:**
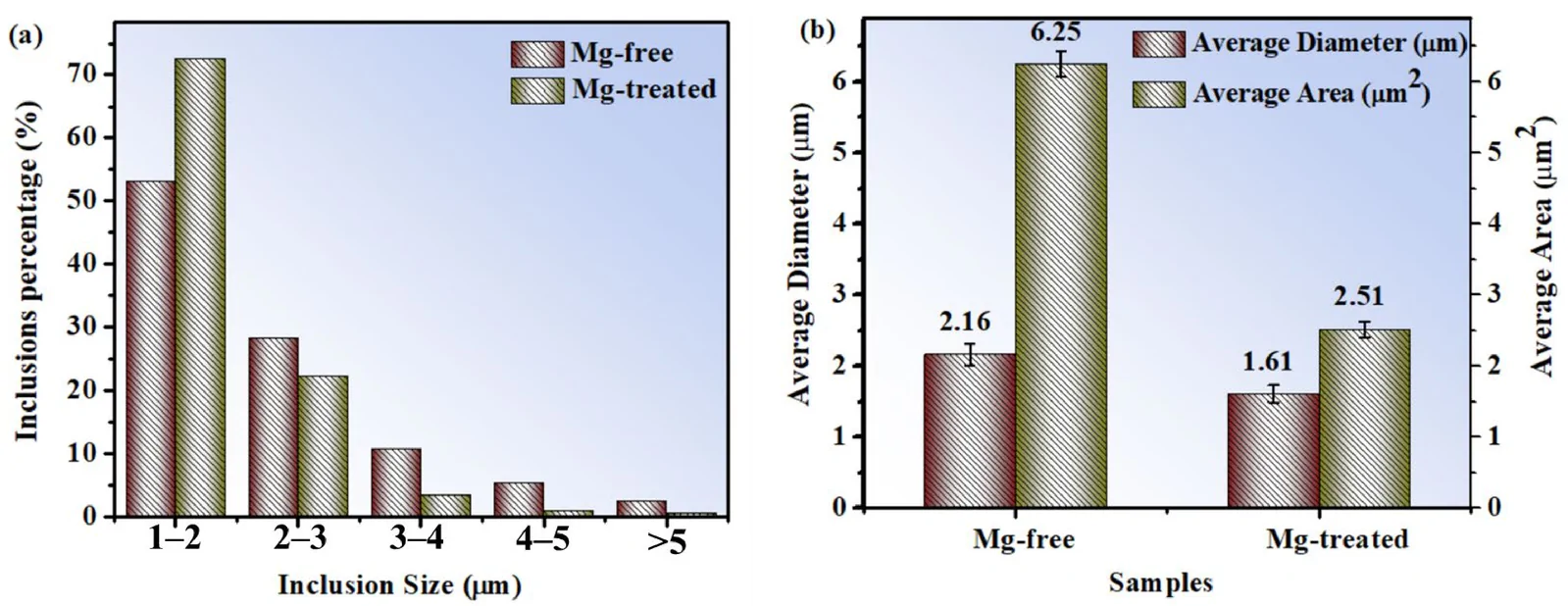
Quantitative analysis of inclusion characteristics: (**a**) Statistical analysis of inclusion size. (**b**) Inclusion dimensions (mean diameter and area) in comparison.

A detailed quantitative assessment of inclusion characteristics, particle size distribution, and morphological parameters was conducted, and the corresponding results are presented in Figure 2. The statistical analysis clearly indicates that Mg addition exerts a significant refining influence on the inclusion population. For the Mg-free steel, the average inclusion diameter was 2.16 μm, with inclusions in the 1–2 μm size interval constituting approximately 53% of the total population, while coarse inclusions larger than 5 μm represented 2.6%. The mean inclusion area was 6.25 μm^2^. Following Mg treatment, notable changes in inclusion characteristics were observed in the Mg-treated steel. The average inclusion diameter decreased to 1.61 μm, and the proportion of inclusions within the 1–2 μm range increased substantially to 72%. Simultaneously, the fraction of inclusions exceeding 5 μm was reduced to only 0.98%, accompanied by a pronounced decrease in average inclusion area to 2.51 μm^2^. The inclusion detection threshold is 0.4 μm ECD. Inclusions of 0.4–1 μm ECD are included in the calculation of average values. Collectively, these findings indicate that addition of Mg suppresses the formation and growth of coarse inclusions while promoting the generation of finer and more uniformly distributed particles.

**Figure 3:**
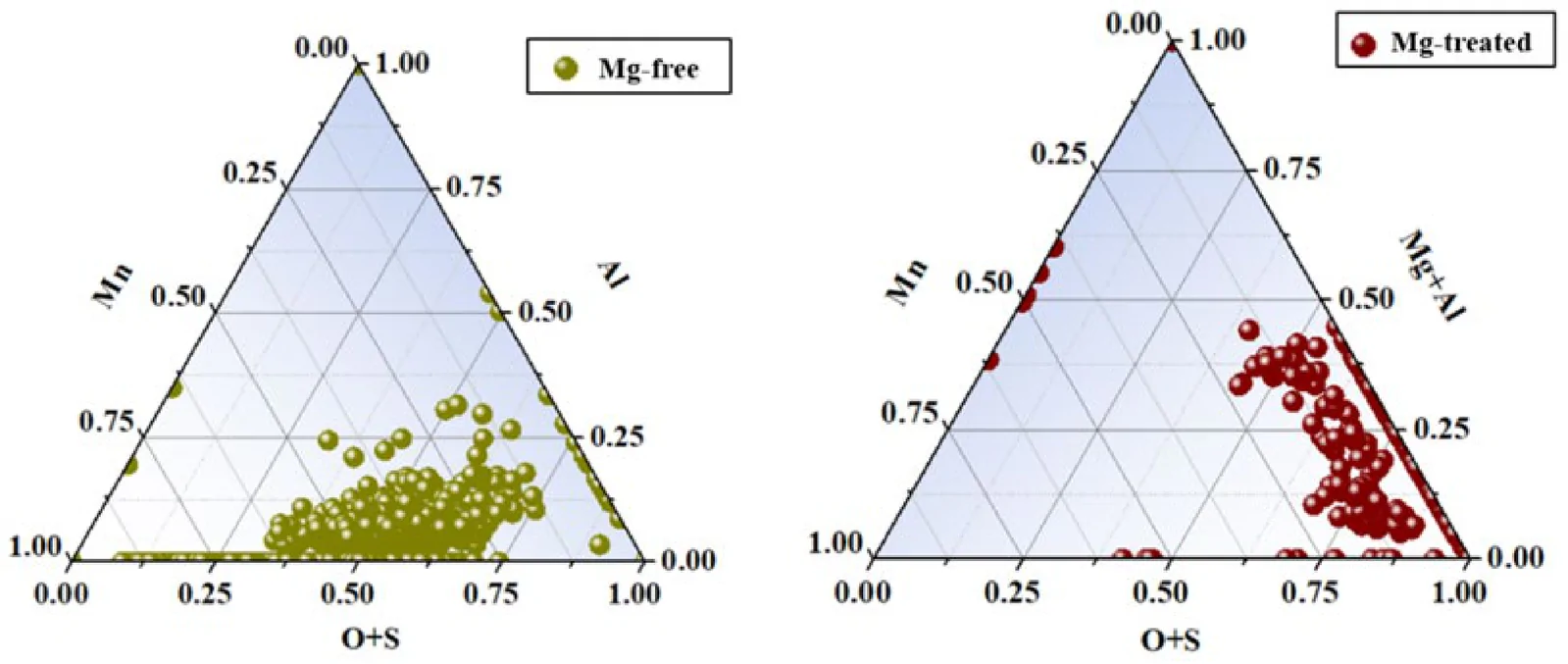
Ternary composition plot showing the weight fraction of non-metallic inclusions detected in Mg-free and Mg-treated steels.

Figure 3 illustrates the ternary compositional distribution of inclusions in Mg-free and Mg-treated steels. In Mg-free steel, inclusions are mainly distributed in the O + S-rich region with relatively low Mn and Al contents, indicating the predominance of (Mn, Al)–O complex and MnS inclusions. After Mg treatment, the inclusion compositions become more concentrated and shift toward the Mg + Al-rich region, while the fraction of Mn-rich inclusions decreases. These results suggest that Mg effectively modifies the original inclusion chemistry by promoting the formation of Mg-containing complex oxides and suppressing Mn-rich inclusions. These observations are consistent with the SEM–EDS analysis, which revealed the transformation of the original Mn–Al oxide inclusions into Mg-containing oxide inclusions accompanied by MnS precipitation at the inclusion boundaries.

### 3.2. Influences of Inclusions on Local Corrosion Responses

#### 3.2.1. Morphological Analysis of Corrosion After Immersion Testing

**Figure 4:**
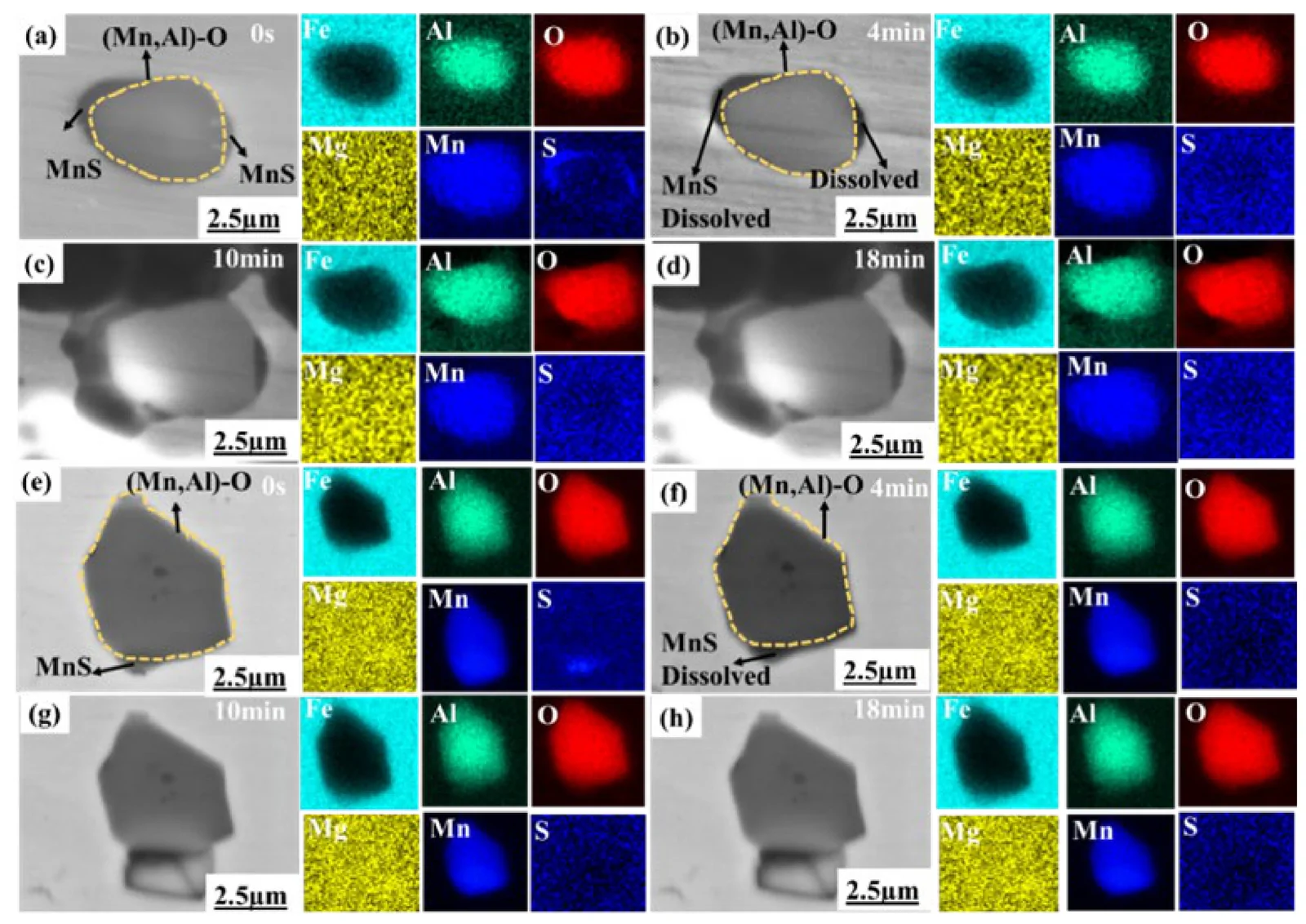
SEM images and corresponding EDS elemental maps showing the time-dependent evolution of two representative non-metallic inclusions in Mg-free steel during immersion for 0, 4, 10, and 18 min in the FeCl_3_·6H_2_O-HCl aqueous solution at 25 °C. (**a**–**d**) First representative inclusion; (**e**–**h**) Second representative inclusion.

Immersion corrosion testing was carried out to investigate the evolution of non-metallic inclusions and their associated localized corrosion behavior. Figure 4 shows the surface morphology of representative composite inclusions in Mg-free steel after different immersion durations of 0 s (as-received condition), 4 min, 10 min, and 18 min. The same individual inclusions were tracked at different immersion stages using pre-established reference marks, enabling their morphological evolution to be directly compared. Initially, the inclusions consisted of composite MnS/(Mn,Al)-O oxides.

After 4 min of immersion, localized dissolution of MnS became evident, whereas the oxide component remained comparatively stable. With increasing immersion time, the MnS-containing regions progressively disappeared, and the morphology of the surrounding steel matrix evolved accordingly. In particular, at 10 min, localized dissolution of the steel matrix was observed in the region adjacent to the MnS, while the same localized surface degradation had progressed further after 18 min. A similar time-dependent evolution was observed for the second representative inclusion (Figure 4e–h), confirming that MnS dissolution and the associated localized matrix dissolution were reproducible features of the immersion process. These observations indicate that preferential dissolution of MnS is closely associated with subsequent localized attack of the neighboring steel matrix. The dissolution of MnS may locally modify the chemical and electrochemical conditions at the inclusion/matrix interface, thereby facilitating localized matrix dissolution and contributing to the development of inclusion-associated corrosion sites.

**Figure 5:**
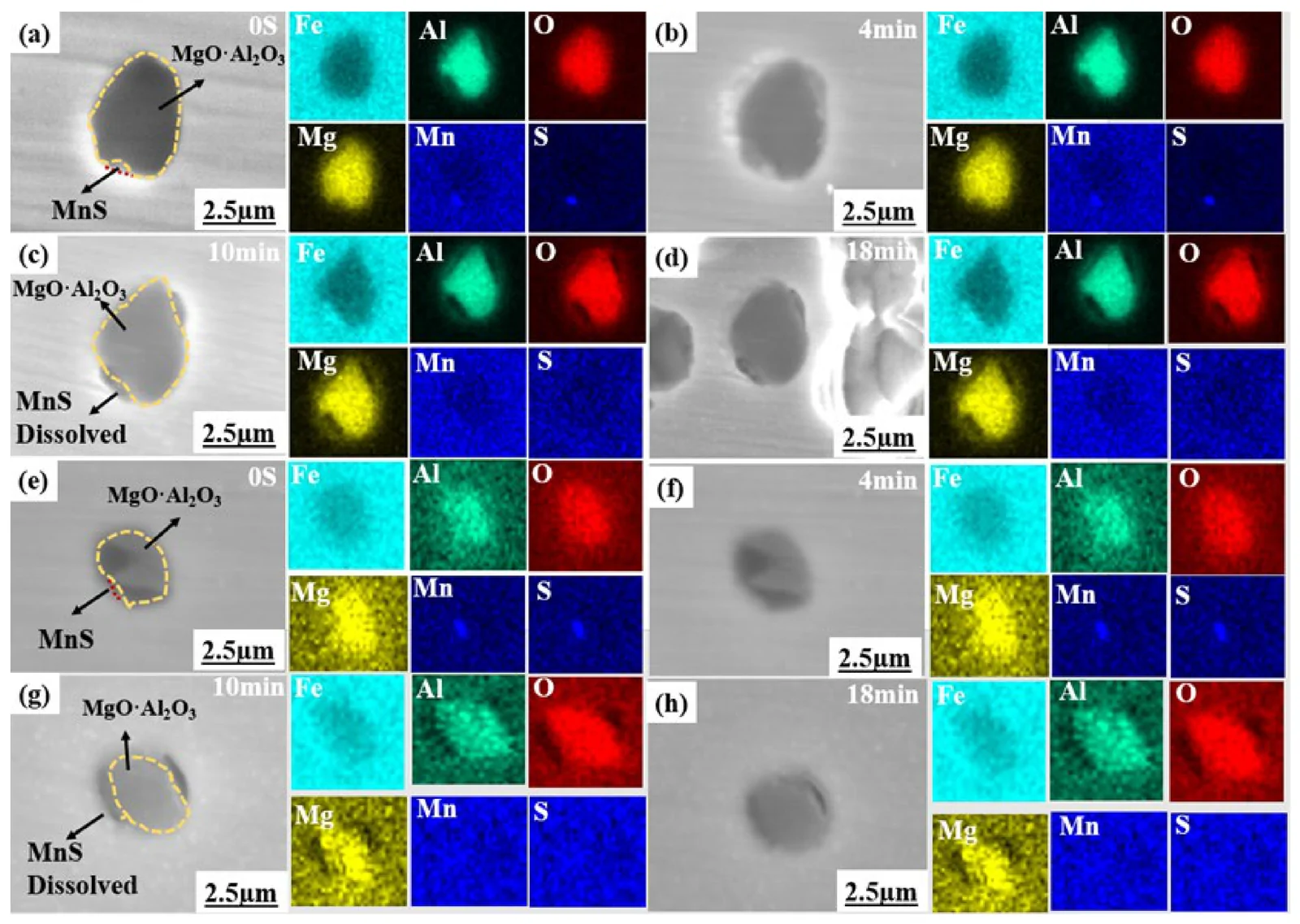
SEM images and corresponding EDS elemental maps showing the time-dependent evolution of two representative non-metallic inclusions in the Mg-treated steel during immersion for 0, 4, 10, and 18 min in the FeCl_3_·6H_2_O-HCl aqueous solution at 25 °C. (**a**–**d**) First representative inclusion. (**e**–**h**) Second representative inclusion.

Figure 5 illustrates the time-dependent evolution of representative non-metallic inclusions in the Mg-treated steel during immersion for 0, 4, 10, and 18 min. Initially, the inclusions consisted of MgO·Al_2_O_3_-rich oxide accompanied by adjacent MnS. With increasing immersion time, the MnS progressively diminished, whereas the MgO·Al_2_O_3_-rich oxide components remained comparatively stable. At 10 min, MnS dissolution was evident, while the subsequent corrosion response differed between the two representative inclusions. For the first inclusion, localized surface corrosion developed during the later immersion stages; however, the corrosion feature was spatially separated from the original MnS dissolution site. In contrast, the second inclusion exhibited MnS dissolution without obvious localized attack of the surrounding steel matrix even after 18 min of immersion. The relatively stable MgO·Al_2_O_3_-rich oxide and the limited development of localized matrix attack around the Mg-treated inclusions indicate that Mg-induced inclusion modification suppresses severe inclusion-associated localized corrosion during immersion.

#### 3.2.2. Analysis of Inclusion-Matrix Potential Distribution via SKPFM Testing

**Figure 6:**
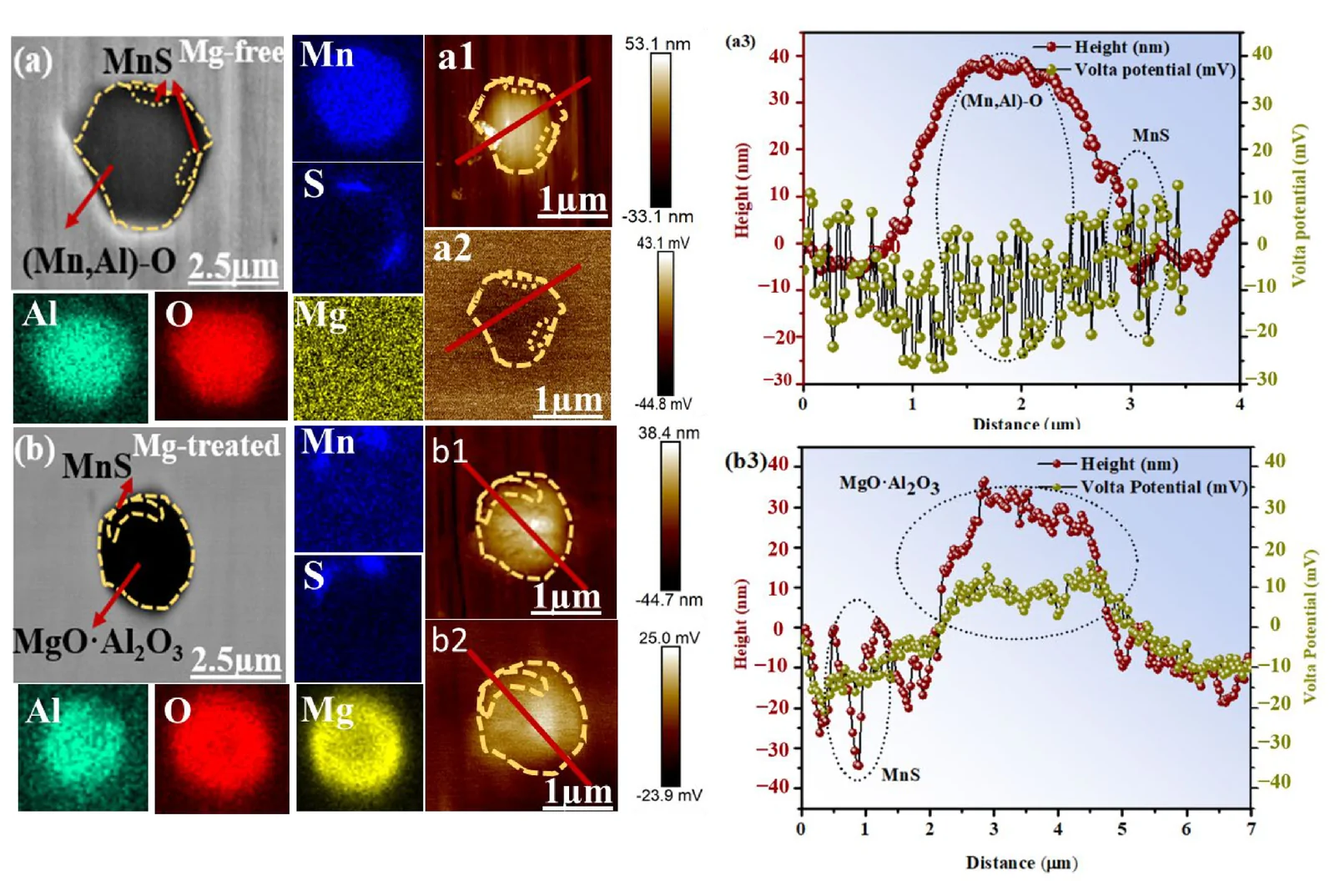
SKPFM characterization of typical non-metallic inclusions in Mg-free and Mg-treated steel. (**a**) SEM micrograph and elemental distribution maps of the inclusion in Mg-free steel; (**a1**,**a2**) SKPFM Volta potential maps of the inclusion in Mg-free steel; (**a3**) corresponding height and Volta potential line-scan profiles. (**b**) SEM micrograph and elemental distribution maps of the inclusion in Mg-treated steel; (**b1**,**b2**) SKPFM Volta potential maps of the inclusion in Mg-treated steel; (**b3**) corresponding height and Volta potential line-scan profiles.

Figure 6 shows the effect of Mg addition on the microelectrochemical properties of representative inclusions. The Volta potential difference measured by SKPFM reflects the local electrochemical heterogeneity between non-metallic inclusions and the surrounding steel matrix. For the Mg-free steel, the composite inclusion consists of (Mn,Al)-O oxide with MnS (Figure 6a). As shown in Figure 6(a3), the entire composite inclusion exhibits a reduced Volta potential relative to the steel matrix. Since the (Mn,Al)-O oxide phase is electrically non-conductive, its direct participation in galvanic corrosion is limited. In contrast, MnS is electrically conductive and has been widely reported to serve as an anodic site relative to the austenitic matrix [25].

Figure 6b shows the Mg-modified inclusions in the Mg-treated steel, mainly composed of MgO·Al_2_O_3_ spinel associated with MnS. The topographical profile reveals that the MgO·Al_2_O_3_ inclusion protrudes approximately 35 nm above the matrix, whereas the MnS region is slightly lower than the surrounding surface. Moreover, the MgO·Al_2_O_3_ phase exhibits a Volta potential approximately 10 mV higher than that of the matrix. The MgO·Al_2_O_3_ spinel is also a relatively stable, poorly conducting oxide and is not expected to behave as an effective metallic galvanic electrode. Compared with the Mg-free steel, the Mg-treated inclusions exhibit a modified volta potential distribution, indicating a change in the local electrochemical heterogeneity associated with inclusion modification. However, the Volta potential measured by SKPFM at room temperature in ambient air does not directly represent the electrode potential or galvanic current in an aqueous electrolyte.

The association of MnS with chemically stable Mg-containing oxides alters the local electrochemical state of composite inclusions. Although MnS remains present after Mg treatment, its contribution to localized corrosion may be influenced by the surrounding oxide phases and the inclusion–matrix interface. This interpretation is consistent with the reduced pitting observed around Mg-modified inclusions during immersion. Multiple individual inclusions were examined for each sample, and the general trend in the volta potential contrast between the inclusions and surrounding matrix was consistently observed, although some quantitative variation occurred among individual inclusions. Accordingly, the SKPFM maps are considered representative observations of local surface-potential heterogeneity, while the proposed relationship between inclusion modification and corrosion resistance is based on the combined SKPFM and immersion results.

### 3.3. Electrochemical Corrosion Performance Analysis

#### 3.3.1. Analysis via Potentiodynamic Polarization Tests

**Figure 7:**
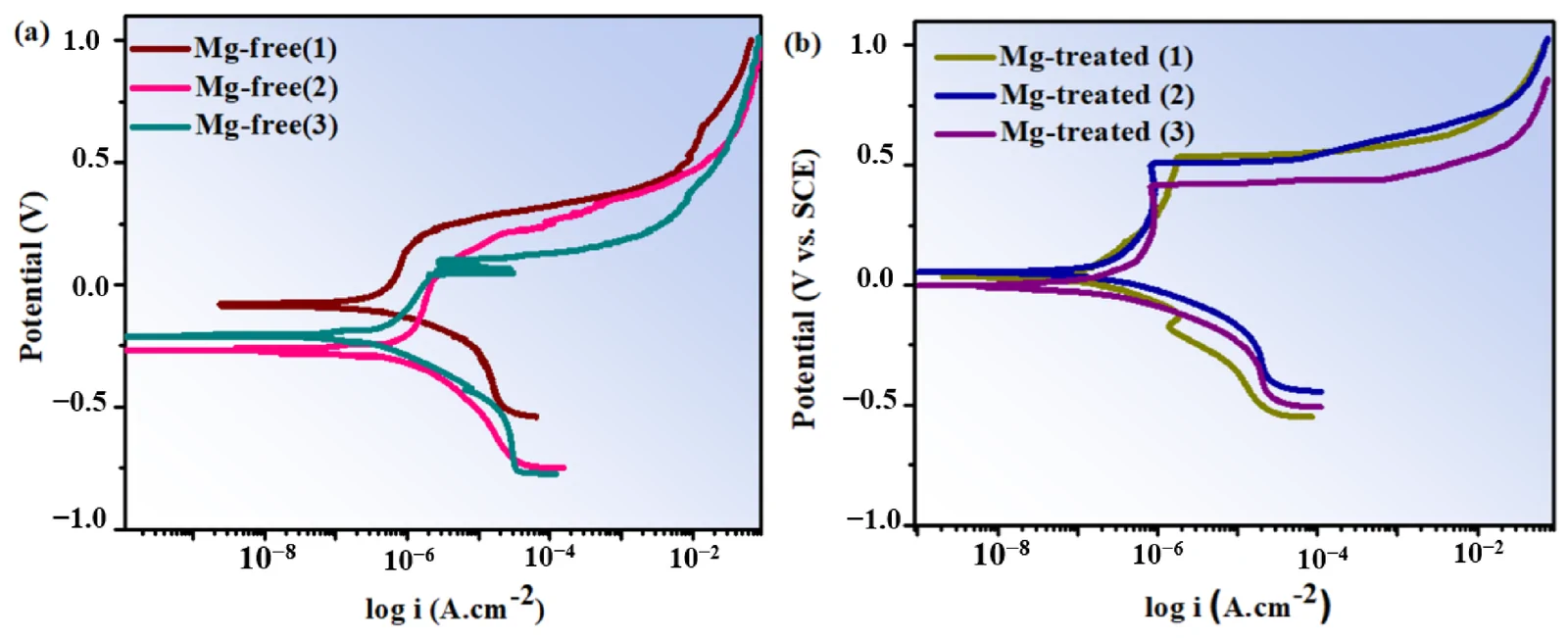
Potentiodynamic polarization curves recorded from (**a**) Mg-free and (**b**) Mg-treated steels immersed in 3.5 wt.% sodium chloride solution under room-temperature conditions.

The potentiodynamic polarization behavior of the Mg-free and Mg-treated steels in 3.5 wt.% NaCl solution at ambient temperature was investigated to evaluate their electrochemical behavior, as shown in Figure 7. The polarization data were analyzed to obtain values of corrosion current density i_corr_, corrosion potential E_corr_, passive current density i_p_ and pitting potential E_pit_; the corresponding parameters are summarized in Table 2. The results indicate that Mg-treated steel exhibits a lower i_corr_, suggesting a reduced corrosion rate. Notably, the passive current density i_p_ decreases from (2.0 ± 0.47) × 10^−6^ A.cm^−2^ for the Mg-free steel to (1.1 ± 0.45) ×10^−6^ A.cm^−2^ for the Mg-treated steel. A lower i_p_ reflects suppressed ionic transport across the passive oxide layer, which confirms the formation of a denser, less defective passive film after Mg modification. More importantly, the E_pit_ value increases substantially from (0.13 ± 0.03) V to (0.47 ± 0.06) V, indicating delayed onset of stable localized corrosion and improved resistance to passive film breakdown. The polarization experiments were performed at least three times for each condition under identical experimental conditions, and the repeated measurements showed consistent polarization behavior. The resulting electrochemical parameters are presented as mean ± standard deviation.

#### 3.3.2. EIS Analysis

**Figure 8:**
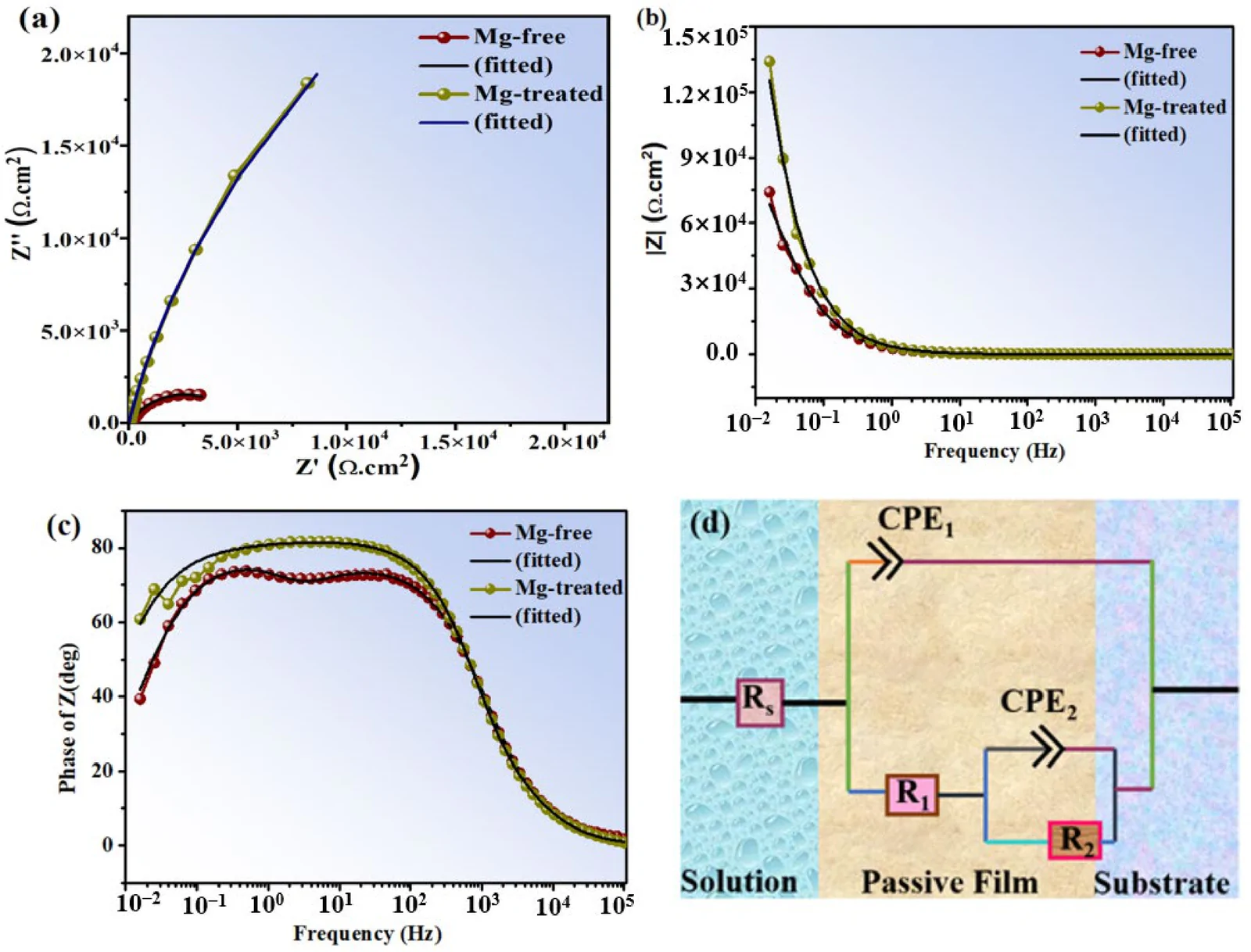
Electrochemical impedance spectroscopy (EIS) results for Mg-free and Mg-treated steels: (**a**) Nyquist Plots, (**b**) Bode impedance Plots, (**c**) Bode theta-angle Plots, and (**d**) equivalent circuit model.

Figure 8 presents the electrochemical impedance spectroscopy (EIS) results of Mg-free and Mg-treated steels in the form of Nyquist and Bode plots. As shown in Figure 8a, both samples exhibit capacitive semicircles characteristic of passive stainless steels in chloride-containing environments. Notably, the diameter of the capacitive arc for the Mg-treated steel is substantially larger than that of the Mg-free steel, indicating that Mg treatment significantly enhances the electrochemical resistance of 304 stainless steel. The enlarged semicircle diameter is associated with increased charge transfer resistance, suggesting suppressed interfacial electrochemical reactions and improved corrosion resistance after Mg addition. The EIS measurements were performed at least three times for each steel condition under identical experimental conditions. The repeated measurements showed consistent Nyquist and Bode responses, confirming the reproducibility of the EIS results.

The Bode plots shown in Figure 8b further confirm this behavior. In the high-frequency region, the impedance modulus mainly reflects the solution resistance, whereas in the low-frequency region it corresponds to the combined contribution of passive film resistance and charge transfer resistance. Compared with the Mg-free steel, the Mg-treated steel displays significantly greater impedance values at low frequencies, indicating enhanced corrosion resistance in the 3.5 wt.% NaCl environment. The higher low-frequency response suggests that magnesium addition hinders electrochemical reactions at the steel/electrolyte interface.

Figure 8c illustrates the phase angle spectra of the investigated steels. The maximum phase angles of the Mg-free and Mg-treated steels are approximately 40° and 60°, respectively. The higher phase angle after Mg addition indicates a more pronounced non-ideal capacitive response of the passive interface, consistent with its enhanced electrochemical resistance.

The EIS data were fitted using the R_s_(Q_film_(R_film_(Q_dl_R_ct_))) equivalent circuit model, as illustrated in Figure 8d. This circuit with two time constants was selected because it provided a satisfactory fit to the experimentally observed impedance spectra. In this model, R_s_ represents the solution resistance, CPE_1_(Q_film_) represents the non-ideal capacitive response associated with the passive-film region, and R_1_(R_film_) corresponds to the resistance of the surface film. R_2_(R_ct_) denotes the charge transfer resistance associated with electrochemical reactions at the metal/electrolyte interface while CPE_2_(Q_dl_) represents the non-ideal interfacial double-layer response.

Two time constants (τ_1_ and τ_2_) were calculated from the fitted EIS parameters using τ = (R. Y_o_)^1/*n*^_,_ to distinguish electrochemical events at medium and low frequencies. τ_1_ is associated with the medium-frequency response of the passive film, while τ_2_ represents the low-frequency time constant related to electrical double-layer formation and interfacial charge-transfer reactions at the steel/electrolyte interface. The Mg-treated steel shows higher values τ_2_ compared with the Mg-free sample. The remarkable increase in τ_2_ for the Mg-treated steel reveals significant retardation of the interfacial charge transfer process and modified double-layer characteristics in the low-frequency domain, confirming that Mg addition suppresses substrate dissolution reactions.

The fitted electrochemical parameters summarized in Table 3 further demonstrate the beneficial effect of Mg addition. The Mg-treated steel exhibits substantially higher R_1_ and R_2_ values compared with the Mg-free steel, indicating enhanced passive film protectiveness and greater resistance to charge transfer processes. In particular, the remarkable increase in charge transfer resistance suggests that Mg treatment effectively suppresses anodic dissolution and slows the overall corrosion kinetics.

### 3.4. XPS Analysis of Passive Film Chemistry

**Figure 9:**
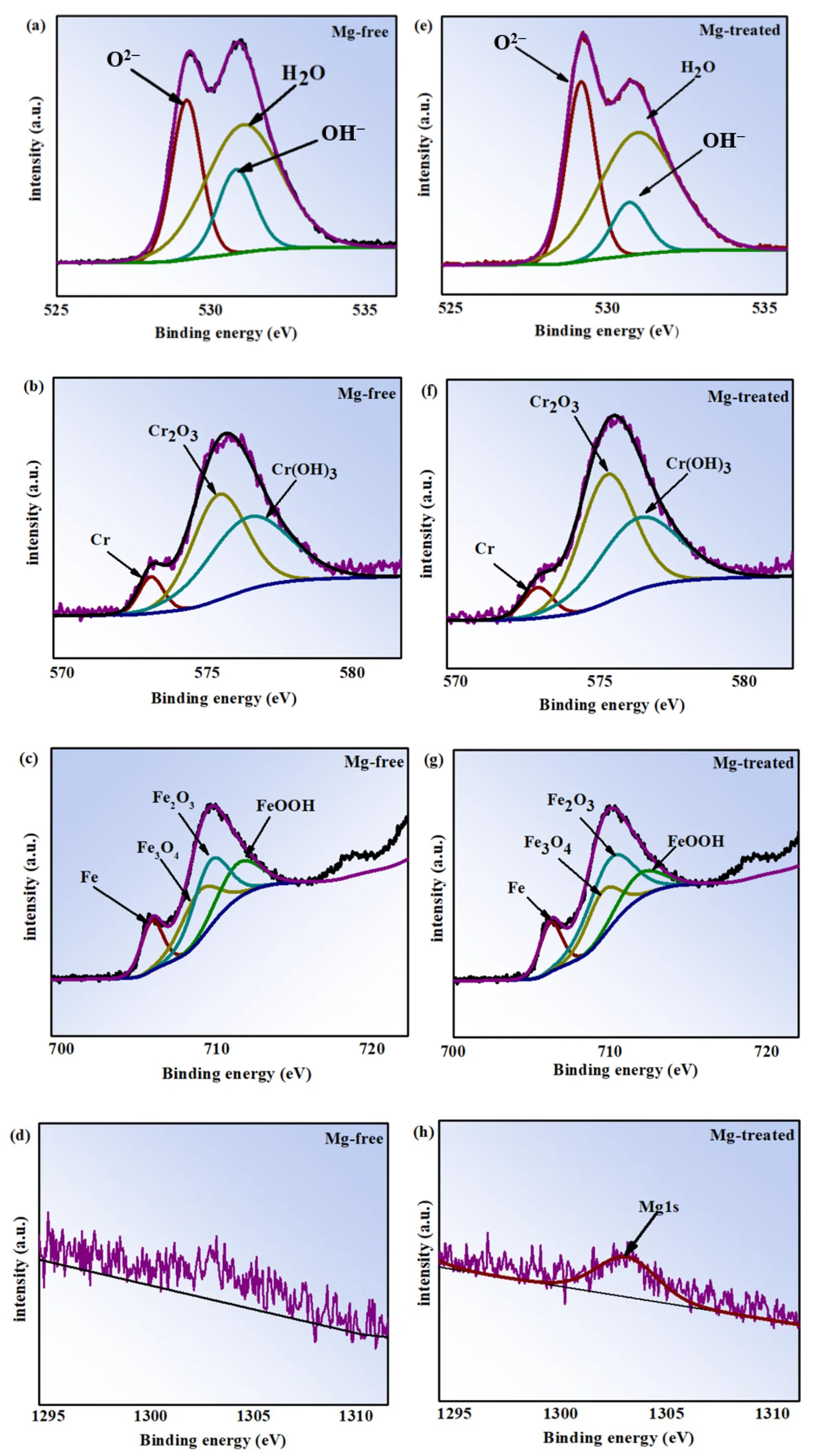
High-resolution XPS spectra of O 1s, Cr 2p, Fe 2p, and Mg 1s for passive films formed in 3.5 wt.% NaCl Solution: (**a**–**d**) Mg-free; (**e**–**h**) Mg-treated.

**Figure 10:**
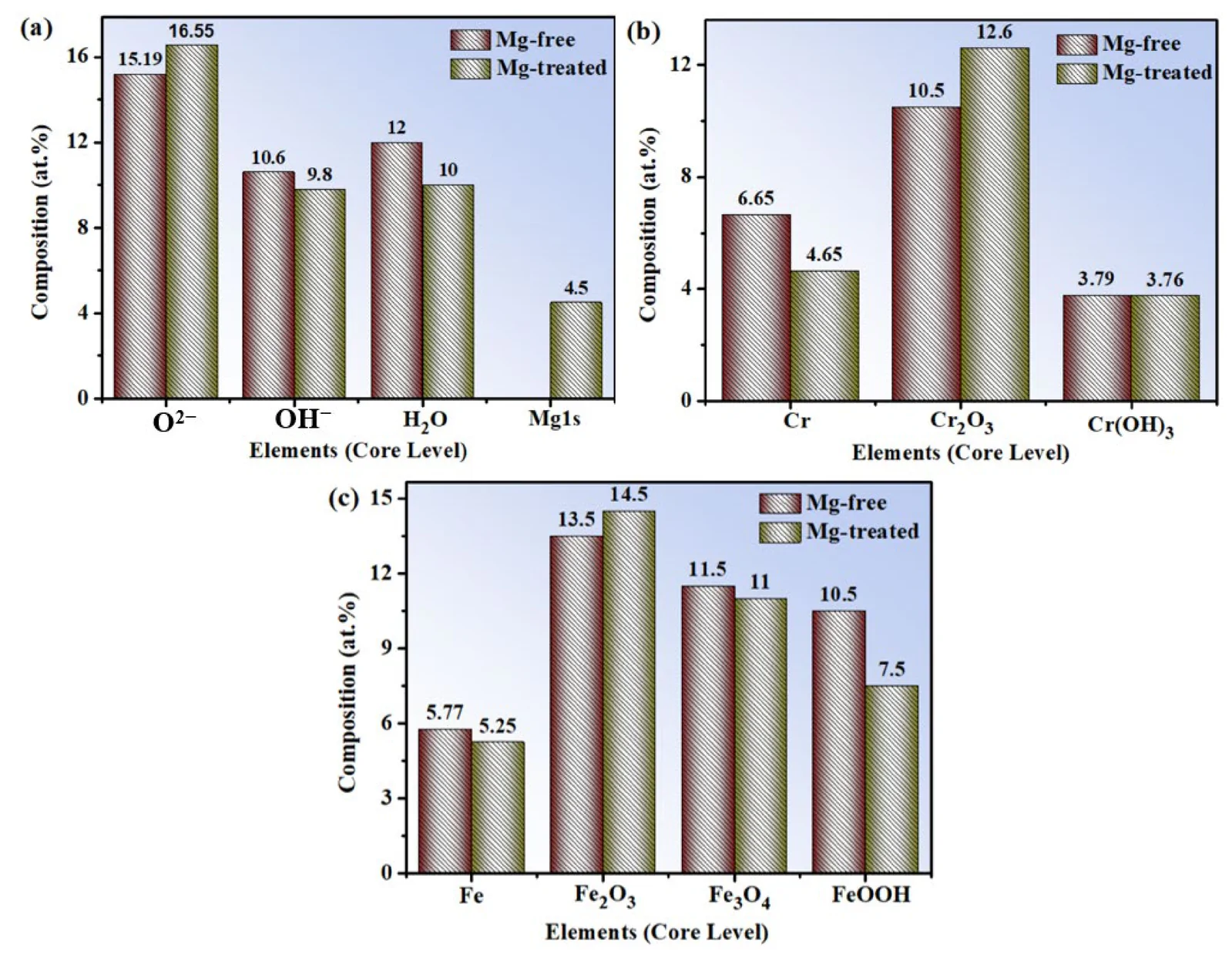
The at. % composition data of (**a**) O 1s, Mg 1s, (**b**) Cr 2p, and (**c**) Fe 2p for the Mg-free and Mg-treated in 3.5 wt.% NaCl solution.

The chemical composition and oxidation states of the passive films formed on Mg-free and Mg-treated samples were analyzed using high-resolution X-ray photoelectron spectroscopy (XPS). The survey spectra and corresponding high-resolution spectra are presented in Figure 9, while the quantitative fitting results for O 1s, Cr 2p, Fe 2p, and Mg 1s are summarized in Figure 10. Atomic percentages of individual chemical states were obtained after relative sensitivity factor (RSF) correction in Avantage and normalized to the sum of the corrected peak areas of all quantified species (O, Cr, Fe, Mg) from the XPS spectra.

The O 1s spectrum was resolved into three components at 529.1 eV, 530.2 eV, and 531.1 eV, assigned to lattice oxide (O^2−^), hydroxide (OH^−^), and adsorbed water (H_2_O), respectively. As shown in Figure 10a the Mg-treated sample exhibits the highest fraction of oxide ions (O^2−^, 16.55%) along with the lowest content of adsorbed water (H_2_O, 10%). The increased O^2−^/OH^−^ ratio (1.68) indicates the formation of a compact and less hydrated passive film, which effectively suppresses electrolyte penetration and enhances resistance to localized corrosion [26]. Such an oxide-rich and compact film structure is widely recognized as more protective in chloride-containing environments. The Mg 1s spectra show a weak but distinct peak at approximately 1303.5 eV for the Mg-treated sample, whereas no resolvable Mg signal is detected for the Mg-free counterpart. This weak feature was distinguished from background noise based on its characteristic binding energy and peak profile, rather than signal intensity alone. This binding energy suggests that Mg exists predominantly in an oxidized state, most likely as MgO. It should be noted that this weak Mg signal may arise either from discrete magnesium-bearing inclusions within the XPS-probed region or from Mg-containing oxide species locally incorporated into the passive film, consistent with the heterogeneous distribution of Mg after inclusion modification. The Cr 2p spectrum was deconvoluted into metallic Cr (574.4 eV), Cr_2_O_3_ (576.3 eV), and Cr (OH)_3_ (577.5 eV). As presented in Figure 10b, the Mg-treated sample exhibits a higher fraction of Cr_2_O_3_ along with reduced amounts of metallic Cr and Cr(OH)_3_. The increased contribution of Cr_2_O_3_ indicates a greater proportion of stable chromium oxide within the passive film. Because Cr_2_O_3_ is associated with the formation of a compact and protective oxide layer, its increased fraction is consistent with improved passive-film stability and resistance to localized corrosion in the chloride-containing environment [27,28].

The Fe 2p spectrum was fitted with metallic Fe (706.3 eV), Fe_3_O_4_ (709.3 eV), Fe_2_O_3_ (710.3 eV), and FeOOH (712.4 eV). Figure 10c shows that the Mg-treated sample contains a higher proportion of Fe_2_O_3_ and a lower fraction of FeOOH. Fe_2_O_3_ and Fe_3_O_4_ are relatively stable oxides that hinder the diffusion of aggressive species toward the substrate, whereas FeOOH is generally associated with less protective and more hydrated corrosion products. Therefore, the observed increase in stable iron oxides and reduction in FeOOH further indicate the formation of a more protective passive film in the Mg-treated sample.

## 4. Discussion

### 4.1. Thermodynamic Analysis of Inclusion Modification via Magnesium Addition

**Figure 11:**
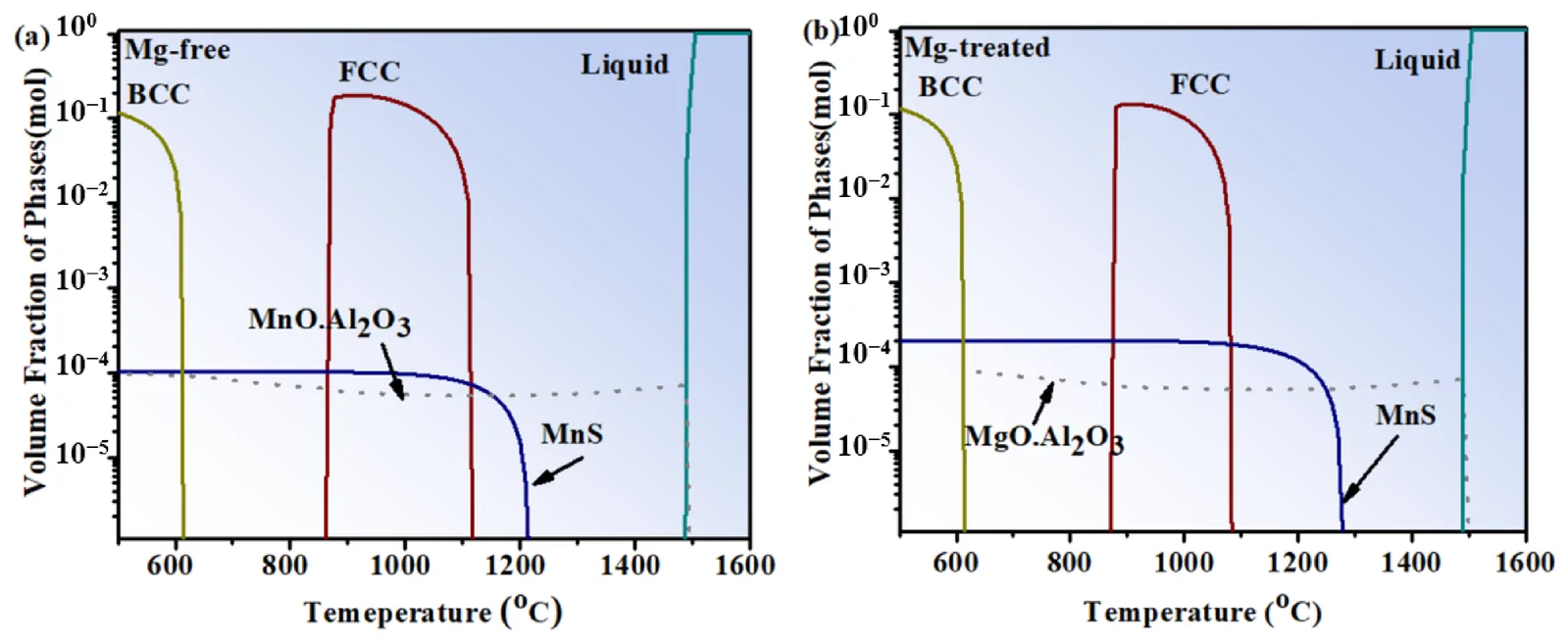
Evolution of inclusions in steel during solidification calculated by Thermo-Calc: (**a**) Mg-free and (**b**) Mg-treated steel.

To explain the role of magnesium in modifying inclusion characteristics, Thermo-Calc equilibrium simulations were performed to predict the precipitation behavior of non-metallic inclusions across the temperature range relevant to solidification (calculation details are provided in Section 2.5). The calculated precipitation sequences for Mg-free and Mg-treated steels are presented in Figure 11. For Mg-free steel, the simulation predicts the formation of MnO·Al_2_O_3_ inclusions during the liquid-solid transformation stage. These oxide inclusions remain thermodynamically stable over a broad temperature range and are accompanied by the precipitation of MnS at lower temperatures (approximately 1200 °C), resulting in the formation of oxide-sulfide composite inclusions. Such inclusions are known to act as preferential sites for localized corrosion initiation because of the electrochemical instability of MnS [29,30].

In contrast, after magnesium addition, the dominant oxide phase changes from MnO·Al_2_O_3_ to MgO·Al_2_O_3_ spinel. The calculated precipitation behavior also indicates a change in the precipitation characteristics of MnS compared with the Mg-free steel. This change, together with the experimentally observed refinement of oxide inclusions and altered MnS morphology, suggests that Mg modifies the inclusion evolution pathway during solidification. This behavior can be attributed to the strong affinity of magnesium for oxygen, which promotes the transformation of Mn-containing oxides into thermodynamically more stable Mg-containing spinel phases. The replacement of MnO·Al_2_O_3_ by fine and stable MgO·Al_2_O_3_ spinel inclusions suppresses the formation of large oxide–sulfide complexes and improves inclusion stability [31]. This thermodynamic prediction is consistent with the SEM-EDS observations presented in Section 3, where Mg-treated steel exhibits refined Mg-containing inclusions with a reduced tendency for MnS decoration. Consequently, magnesium-induced inclusion modification is expected to mitigate micro-galvanic effects between inclusions and the steel matrix, thereby contributing to the enhanced pitting corrosion resistance observed in the electrochemical tests.

### 4.2. Influence of Mg Addition on Inclusion-Associated Localized Corrosion

Non-metallic inclusions, particularly MnS-containing inclusions, are recognized as preferential sites for localized corrosion initiation in austenitic stainless steels. The inclusion–matrix interface represents a region of chemical and electrochemical heterogeneity where preferential dissolution of MnS can expose the adjacent steel matrix and generate a localized interfacial region susceptible to further corrosion [32]. Previous microelectrochemical studies have demonstrated that MnS dissolution can produce trenching or microcrevice formation along the MnS/matrix boundary, providing a favorable site for subsequent pit initiation [33]. The extent of localized corrosion, however, depends strongly on the inclusion morphology, inclusion–matrix geometry, and local chemical environment [34].

In an acidic environment, the chemical dissolution of MnS can be represented as:

(1) $$MnS+H^{+}\to {Mn}^{2+}+HS^{-}$$

(2) $$S^{-}\to S+H^{+}+2e^{-}$$

The sulfur-containing species released during MnS dissolution, together with the presence of Cl^−^, contribute to an aggressive localized environment that promotes dissolution at the MnS/matrix boundary. The confined geometry and ongoing anodic dissolution can promote local acidification, thereby favoring further dissolution and pit stabilization.

In contrast, steels subjected to magnesium treatment display a substantially revised mechanism. The Mg addition modifies the nature, morphology, and spatial distribution of MnS-containing inclusions, leading to the development of more thermodynamically stable Mg-containing oxide particles as supported by the Thermo-Calc calculations. The association of MnS with Mg-modified oxide inclusions reduces the fraction of sulfide phase directly exposed to the electrolyte. The modified inclusion architecture appears to reduce the tendency for MnS dissolution to develop into pronounced localized attack of the surrounding steel matrix.

The modified volta potential distribution observed around the Mg-containing inclusions suggests changes in local electrochemical heterogeneity between the inclusions and the surrounding steel matrix. Together with the altered inclusion morphology and reduced exposure of MnS, this modified electrochemical environment may contribute to the suppressed development of inclusion-associated localized corrosion. Overall, the improved corrosion resistance of the Mg-treated steel can be attributed to the combined suppression of electrochemical driving forces for inclusion dissolution and disruption of the localized chemical conditions required for sustained pitting propagation. In addition to the intentional Mg addition (0.003 wt.%), the two steels differ slightly in S (0.002 vs. 0.003 wt.%), C, Mn, and Si contents. Since Mg was added during industrial ladle furnace refining, fully independent heat replication with an identical controlled S matrix was not feasible. The 0.001 wt.% difference in S is small, and both alloys remain in the low-S regime (<0.005 wt.%). The observed inclusion modification (MnS-associated (Mn,Al)-O → MnS-decorated MgO·Al_2_O_3_) is consistent with the thermodynamic affinity of Mg for oxygen, as supported by the Thermo-Calc calculations in Section 4.1. While subtle effects from these minor compositional offsets cannot be entirely excluded, the dominant driving force for inclusion evolution is attributed to Mg addition.

**Figure 12:**
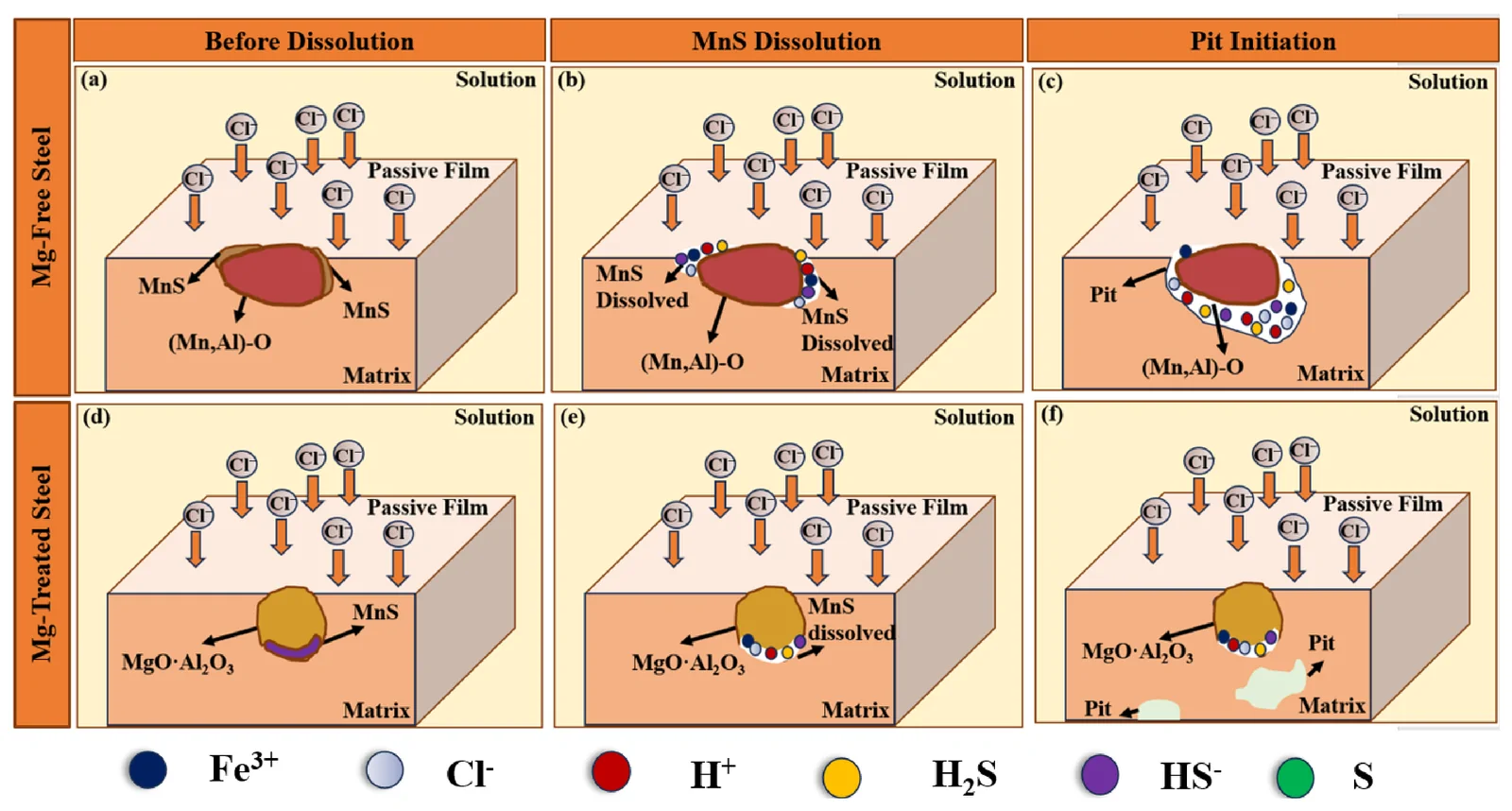
Schematic illustration of the proposed mechanism governing pathways for the onset and growth of pitting corrosion in the (**a**–**c**) Mg-free steel and (**d**–**f**) Mg-treated steel.

A schematic illustration of the MnS-driven localized corrosion mechanism and its modification by Mg addition is presented in Figure 12.

### 4.3. Role of Magnesium Addition in Tailoring the Corrosion Performance and Passive Film Stabilization of 304 Austenitic Stainless Steel

The electrochemical measurements demonstrate that Mg addition improves the corrosion resistance of 304 stainless steel in 3.5 wt.% NaCl solution. Potentiodynamic polarization measurements (Figure 7, Table 2) reveal a pronounced noble shift in the pitting potential (E_pit_) from (0.13 ± 0.04) V to (0.47 ± 0.05) V (vs. SCE) upon Mg addition, accompanied by a reduction in corrosion current density (i_corr_) from 0.50 × 10^−6^ A·cm^−2^ to 0.17 × 10^−6^ A·cm^−2^. The passive current density i_p_ is a key descriptor for the protective performance of the passive film. The Mg-treated steel shows a reduced passive current density (1.1 ± 0.45) × 10^−6^ A.cm^−2^ compared with the Mg-free sample (2.0 ± 0.47) × 10^−6^ A.cm^−2^. This reduction in i_p_ indicates that Mg incorporation modifies the passive film microstructure, decreasing its ionic conductivity and inhibiting the transport of corrosive species through the oxide layer. The higher pitting potential (Ep ≈ 0.47 ± 0.05 V vs. SCE) confirms that Mg addition effectively delays passive film breakdown and improves resistance to chloride-induced localized corrosion [35]. The decrease in i_corr_ further suggests a reduction in the overall corrosion rate under the tested conditions [36].

Electrochemical impedance spectroscopy provides further evidence of the enhanced protective characteristics of the Mg-modified surface. As summarized in Table 3, the charge transfer resistance (R_2_) increases by more than one order of magnitude from 1.07 ± 0.13 × 10^5^ Ω·cm^2^ to 3.5 ± 0.13 × 10^5^ Ω·cm^2^ after Mg addition, indicating a substantially suppressed charge transfer process at the metal/electrolyte interface [37]. In parallel, the increase in film resistance (R_1_) from 4338 ± 0.13 Ω·cm^2^ to 6270 ± 0.11 Ω·cm^2^ suggests improved barrier properties of surface film and greater resistance to interfacial transport processes [38].In addition, the maximum phase angle increases from approximately 40° for the Mg-free steel to 60° for the Mg-treated steel. The higher phase angle indicates a more capacitive and relatively stable passive layer, suggesting enhanced film integrity and surface homogeneity [39,40].

The changes in electrochemical behavior are accompanied by modifications in the surface-film chemistry, as revealed by XPS analysis performed at 0.1 V in 3.5 wt.% NaCl solution. The XPS results indicate the presence of Mg-containing surface species and changes in the O 1s spectral components following Mg addition. These observations suggest that Mg addition influences the chemical composition of the surface film. However, the detection of Mg-containing species does not, by itself, establish that Mg is uniformly distributed throughout the passive film or that a continuous MgO layer has formed. Possible reactions associated with Mg-containing surface species and the formation of oxide/hydroxide species during passivation can be represented by the following equations:

(3) $$O_{2\;}+2H_{2}O+4e^{-}\;\to 4OH^{-}$$

Meanwhile, Mg atoms near the surface can undergo anodic dissolution:

(4) $$\mathrm{Mg}\to {Mg}^{2+}+2e^{-}$$

The released Mg^2+^ ions subsequently react with hydroxide ions to form magnesium hydroxide:

(5) $${Mg}^{2+}+2OH^{-}\to Mg\;{\left(OH\right)}_{2}$$

Mg (OH)_2_ may further dehydrate to form MgO

(6) $$Mg\;{\left(OH\right)}_{2}\to MgO+H_{2}O$$

The presence of Mg-containing surface species may contribute to changes in the chemical and electrochemical characteristics of the passive surface. Their possible effects on local interfacial pH, chloride interaction, and transport through the surface film require further direct investigation.

Since the corrosion resistance of stainless steel is fundamentally governed by the stability of the Cr_2_O_3_-rich passive film, maintaining a high concentration of Cr^3+^ species within the oxide layer is essential for effective self-healing after local film breakdown. It is proposed that Mg-containing oxides may stabilize the passive film structure, enhance adhesion between the film and substrate, and reduce defect density. If such changes occur, the rapid reformation of a Cr_2_O_3_-rich protective layer could be facilitated after local disruption of the passive film by aggressive chloride ions. Overall, Mg addition improves corrosion resistance through a synergistic mechanism involving both inclusion modification and passive film stabilization. On one hand, Mg alters the inclusion chemistry, reducing the detrimental effect of MnS inclusions on pit initiation. On the other hand, Mg may participate in passive film formation, promoting the development of MgO that could enhance film compactness, suppress ionic transport, and stabilize the Cr_2_O_3_-dominated passive layer. These combined effects plausibly enhance the resistance of Mg-alloyed stainless steel to chloride-induced localized corrosion.

## 5. Conclusions

This study systematically investigated the influence of magnesium microalloying on inclusion evolution and localized corrosion behavior of 304 austenitic stainless steel. The main conclusions are as follows:

1. Magnesium addition transformed conventional (Mn,Al)-O inclusions surrounded by MnS into thermodynamically stable MgO·Al_2_O_3_ spinel inclusions. This inclusion modification significantly refined the inclusion population, reducing both inclusion size and the fraction of coarse inclusions.
2. Mg treatment altered the electrochemical and corrosion response of inclusion/matrix regions. In the Mg-free steel, progressive MnS dissolution was accompanied by localized attack of the adjacent matrix. In the Mg-treated steel, MnS dissolution was still observed, but the subsequent localized matrix attack was limited and was not consistently associated with the original MnS dissolution sites. These observations indicate that Mg-induced inclusion modification reduces the tendency for inclusion dissolution to develop into sustained localized matrix attack.
3. Electrochemical measurements in 3.5 wt.% NaCl demonstrated improved corrosion resistance after Mg treatment. The pitting potential increased from (0.13 ± 0.04) V to (0.47 ± 0.05) V (vs. SCE), while the corrosion current density decreased from 0.50 × 10^−6^ to 0.17 × 10^−6^ A·cm^−2^. EIS measurements further showed increased R1 and R2, indicating a more resistive steel/electrolyte interface for the Mg-treated steel.
4. XPS analysis further indicated that Mg addition modified the chemical composition of the surface film. The presence of Mg and O-containing species in the Mg-treated steel suggests the incorporation of Mg-containing oxygen species into the surface film. This modification is consistent with the enhanced barrier characteristics observed from the electrochemical measurements.

Overall, magnesium addition improves the localized corrosion resistance of 304 stainless steel by modifying inclusion composition and morphology, reducing inclusion-associated electrochemical heterogeneity, and suppressing the transition from MnS dissolution to sustained localized matrix attack. These findings provide mechanistic insight into the relationship between inclusion characteristics and corrosion behavior and offer a practical strategy for the design and industrial production of corrosion-resistant stainless steels for chloride-containing service environments.

## Figures and Tables

**Table 1:** Chemical composition of the test steels (wt.%).

Sample	C	Mn	Al	P	S	Si	Cr	Ni	Mg
Mg-free	0.027	0.864	0.004	0.024	0.002	0.345	18.064	8.113	-
Mg-treated	0.023	0.906	0.005	0.022	0.003	0.373	18.063	8.018	0.003

**Table 2:** Electrochemical parameters obtained from potentiodynamic polarization measurements of Mg-free and Mg-treated steels in 3.5 wt.% NaCl solution.

Sample	i_corr_ (A.cm^−2^)	E_corr_ (V)	i_p_ (A.cm^−2^)	E_pit_ (V)
Mg-free	(0.50 ± 0.06) × 10^−6^	−0.19 ± 0.10	(2.0 ± 0.47) × 10^−6^	0.13 ± 0.04
Mg-treated	(0.17 ± 0.04) × 10^−6^	0.04 ± 0.006	(1.1 ± 0.45) × 10^−6^	0.47 ± 0.05

**Table 3:** Electrochemical impedance parameters.

	Mg-Free	Mg-Treated
**R_s_(Ω.cm^2^) (solution resistance)**	6.7 ± 0.12	7.4 ± 0.11
**Y_o1_ (Ω^−1^.cm^−2^)**	6.31 ± 0.07 × 10^−5^	1.6 ± 0.04 × 10^−5^
**n_1_**	0.8 ± 0.006	0.9 ± 0.006
**R_1_(Ω.cm^2^) (Passive resistance)**	4338 ± 0.13	6270 ± 0.11
**Y_o2_ (Ω^−1^.cm^−2^)**	3.9 ± 0.08 × 10^−5^	1.2 ± 0.05 × 10^−5^
**n_2_**	0.9 ± 0.004	0.8 ± 0.002
**R_2_ (Ω.cm^2^) (charge transfer resistance)**	1.07 ± 0.13 × 10^5^	3.5 ± 0.13 × 10^5^
**τ_1_**	0.198 ± 0.0004	0.078 ± 0.02
**τ_2_**	4.891 ± 0.84	6.013 ± 0.64
**Σχ2**	6.5 ± 0.18 × 10^−4^	4.0 ± 0.11 × 10^−4^

## Data Availability

The original contributions presented in this study are included in the article/Supplementary Materials. Further inquiries can be directed to the corresponding authors.

## Supplementary Materials

The following supporting information can be downloaded at: https://www.mdpi.com/article/10.3390/ma19214528/s1, Table S1: XPS peak fitting parameters of Mg-free and Mg-treated samples.
