# Supplementary material for: Influence of Trace Magnesium Addition on Inclusion Modification and Localized Corrosion Behavior of 304 Austenitic Stainless Steel
Source: Materials (Basel). 2026 Oct 27;19(21):4528. doi: 10.3390/ma19214528 (PMC13763945; doi:10.3390/ma19214528)
Supplement: Supplementary file 1 [file materials-19-04528-s001.zip › materials-4619949-supplementary.pdf]

Table S1: XPS peak fitting parameters of Mg-free and Mg-treated samples.

Chemical State	Mg-free		Mg-treated	
	**Binding energy(eV)**	**FWHM(eV)**	**Binding energy(eV)**	**FWHM(eV)**
O^{2-}	529.1	1.1	529.1	1.1
OH^-	530.2eV	1.7	530.2eV	1.4
H_2O	531.1	2.8	531.1	2.8
**Cr**	574.4	1.2	574.4	1.1
Cr_2O_3	576.3	2.2	576.3	2.2
$Cr(OH)_3$	577.5	3.3	577.5	3.3
**Fe**	706.3	1.0	706.3	1.0
Fe_3O_4	709.3	1.3	709.3	1.4
Fe_2O_3	710.3	1.7	710.3	1.8
**FeOOH**	712.4	1.2	712.4	1.4
**Mg1s**	1303.5	3.3	1303.5	3.3
